# Supplementary material for: Sodicity stress differently influences physiological traits and anti-oxidant enzymes in pear and peach cultivars
Source: PeerJ. 2023 Mar 13;11:e14947. doi: 10.7717/peerj.14947 (PMC10019333; doi:10.7717/peerj.14947)
Supplement: Supplemental Information 1 [file peerj-11-14947-s001.docx]

**Table S1** Soil saturation paste pH (pH_s_) and soil saturation extract electrical conductivity (EC_e_) across depth in the experimental field.

| **Treatment** | **Depth (cm)** | **pHs** | **ECe** |
| --- | --- | --- | --- |
| Control | 0-15 | 8.02±0.22d | 0.48±0.09c |
|  | 15-30 | 8.19±0.21cd | 0.64±0.11bc |
|  | 30-60 | 8.24±0.21cd | 0.84±0.13b |
|  | 60-100 | 8.41±0.37bc | 0.82±0.10b |
| Sodic | 0-15 | 8.36±0.24bc | 0.69±0.10b |
|  | 15-30 | 8.57±0.25b | 0.81±0.10b |
|  | 30-60 | 9.09±0.21a | 1.11±0.14a |
|  | 60-100 | 9.16±0.40a | 1.16±0.21a |
|  | *F* | 41.94 | 27.52 |
|  | *p* | ˂0.001 | ˂0.001 |

Note: Each value represents mean ± SD of three replicates. The means in each row having one letter in common are not significantly different according to Tukey’s test (p 0.05).

**Table S2** Variable contributions (%), Eigen values, variance (%) and cumulative variance (%) explained by the first five Dimensions/Principal Components (PCs) in pear and peach.

| **Variable/Dimension** | **Dim.1** | **Dim.2** | **Dim.3** | **Dim.4** | **Dim.5** | **Dim.1** | **Dim.2** | **Dim.3** | **Dim.4** | **Dim.5** |
| --- | --- | --- | --- | --- | --- | --- | --- | --- | --- | --- |
|  | Pear |  |  |  |  | Peach |  |  |  |  |
| TH | 5.60 | 11.03 | 0.37 | 1.24 | 0.54 | 11.01 | 1.67 | 1.72 | 7.42 | 2.31 |
| TCSA | 4.05 | 13.43 | 1.14 | 1.24 | 0.10 | 3.28 | 11.06 | 34.00 | 9.68 | 1.77 |
| CV | 8.52 | 2.86 | 14.11 | 31.05 | 8.35 | 9.19 | 4.86 | 0.09 | 2.82 | 1.86 |
| TC | 7.44 | 4.47 | 18.36 | 17.02 | 28.15 | 7.83 | 5.13 | 19.99 | 20.01 | 5.81 |
| MDA | 4.59 | 11.69 | 3.69 | 4.67 | 15.85 | 9.61 | 2.94 | 3.95 | 24.40 | 24.41 |
| H_2_O_2_ | 2.04 | 16.84 | 0.20 | 1.52 | 6.15 | 11.92 | 0.38 | 0.71 | 0.49 | 26.25 |
| Prol | 9.36 | 0.65 | 31.42 | 9.24 | 1.34 | 12.19 | 0.34 | 2.47 | 0.99 | 18.06 |
| APX | 11.75 | 0.30 | 0.09 | 7.61 | 0.69 | 0.13 | 17.83 | 1.54 | 0.57 | 0.27 |
| POX | 7.21 | 6.71 | 6.47 | 0.88 | 28.32 | 0.01 | 17.86 | 2.86 | 0.18 | 3.99 |
| CAT | 4.41 | 12.94 | 0.01 | 0.25 | 6.72 | 3.81 | 12.64 | 1.04 | 1.66 | 0.24 |
| SOD | 6.27 | 8.56 | 4.75 | 3.71 | 0.56 | 9.37 | 3.51 | 8.77 | 24.58 | 7.19 |
| Na | 8.70 | 5.20 | 3.83 | 1.16 | 0.12 | 7.49 | 7.24 | 2.17 | 1.63 | 0.57 |
| K | 8.74 | 5.29 | 1.38 | 12.10 | 0.40 | 4.34 | 11.48 | 4.34 | 3.14 | 7.27 |
| Na.K | 11.30 | 0.03 | 14.21 | 8.30 | 2.70 | 9.82 | 3.05 | 16.35 | 2.42 | 0.01 |
| Eigen value | 7.89 | 4.84 | 0.52 | 0.29 | 0.14 | 7.74 | 5.50 | 0.35 | 0.15 | 0.10 |
| Variance (%) | 56.35 | 34.60 | 3.71 | 2.07 | 1.03 | 55.32 | 39.29 | 2.48 | 1.06 | 0.74 |
| Cumulative (%) | 56.35 | 90.95 | 94.66 | 96.73 | 97.76 | 55.32 | 94.61 | 97.09 | 98.15 | 98.89 |

Abbreviations: TH- Tree height, CV- canopy volume, TCSA- Trunk cross sectional area, TC- Total leaf chlorophyll, MDA- Malondialdehyde, H2O2- Hydrogen peroxide (H_2_O_2_), Prol- Proline, APX- ascorbate peroxidase, POX- Peroxidase, CAT- Catalase, SOD- Superoxide dismutase, Na- Leaf Na^+^, K- Leaf K^+^, Na.K- Leaf Na/K ratio

**Table S3** Pearson's correlation coefficients (*r*) and the associated p-values (*p*) between the measured traits in pear.

| **Trait** | **Trait** | **r** | **p** | **Trait** | **Trait** | **r** | **p** |
| --- | --- | --- | --- | --- | --- | --- | --- |
| TH | TCSA | 0.969 | 0.000 | TCSA | SOD | 0.122 | 0.654 |
| TH | CV | 0.816 | 0.000 | CV | SOD | -0.322 | 0.223 |
| TCSA | CV | 0.802 | 0.000 | TC | SOD | -0.303 | 0.253 |
| TH | TC | 0.825 | 0.000 | MDA | SOD | 0.886 | 0.000 |
| TCSA | TC | 0.786 | 0.000 | H_2_O_2_ | SOD | 0.857 | 0.000 |
| CV | TC | 0.756 | 0.001 | Prol | SOD | 0.755 | 0.001 |
| TH | MDA | 0.146 | 0.591 | APX | SOD | 0.727 | 0.001 |
| TCSA | MDA | 0.243 | 0.364 | POX | SOD | 0.187 | 0.488 |
| CV | MDA | -0.270 | 0.312 | CAT | SOD | 0.919 | 0.000 |
| TC | MDA | -0.121 | 0.655 | TH | Na | -0.200 | 0.457 |
| TH | H_2_O_2_ | 0.396 | 0.129 | TCSA | Na | -0.061 | 0.822 |
| TCSA | H_2_O_2_ | 0.504 | 0.046 | CV | Na | -0.512 | 0.042 |
| CV | H_2_O_2_ | -0.020 | 0.943 | TC | Na | -0.353 | 0.180 |
| TC | H_2_O_2_ | 0.105 | 0.700 | MDA | Na | 0.874 | 0.000 |
| MDA | H_2_O_2_ | 0.914 | 0.000 | H_2_O_2_ | Na | 0.777 | 0.000 |
| TH | Prol | -0.452 | 0.079 | Prol | Na | 0.753 | 0.001 |
| TCSA | Prol | -0.310 | 0.242 | APX | Na | 0.855 | 0.000 |
| CV | Prol | -0.504 | 0.047 | POX | Na | 0.315 | 0.235 |
| TC | Prol | -0.643 | 0.007 | CAT | Na | 0.880 | 0.000 |
| MDA | Prol | 0.578 | 0.019 | SOD | Na | 0.871 | 0.000 |
| H_2_O_2_ | Prol | 0.519 | 0.039 | TH | K | 0.934 | 0.000 |
| TH | APX | -0.542 | 0.030 | TCSA | K | 0.871 | 0.000 |
| TCSA | APX | -0.435 | 0.092 | CV | K | 0.836 | 0.000 |
| CV | APX | -0.693 | 0.003 | TC | K | 0.806 | 0.000 |
| TC | APX | -0.656 | 0.006 | MDA | K | -0.109 | 0.687 |
| MDA | APX | 0.667 | 0.005 | H_2_O_2_ | K | 0.141 | 0.602 |
| H_2_O_2_ | APX | 0.481 | 0.059 | Prol | K | -0.615 | 0.011 |
| Prol | APX | 0.839 | 0.000 | APX | K | -0.773 | 0.000 |
| TH | POX | -0.884 | 0.000 | POX | K | -0.882 | 0.000 |
| TCSA | POX | -0.869 | 0.000 | CAT | K | -0.082 | 0.762 |
| CV | POX | -0.802 | 0.000 | SOD | K | -0.238 | 0.374 |
| TC | POX | -0.881 | 0.000 | Na | K | -0.444 | 0.085 |
| MDA | POX | -0.025 | 0.927 | TH | Na.K | -0.623 | 0.010 |
| H_2_O_2_ | POX | -0.189 | 0.484 | TCSA | Na.K | -0.512 | 0.043 |
| Prol | POX | 0.592 | 0.016 | CV | Na.K | -0.778 | 0.000 |
| APX | POX | 0.662 | 0.005 | TC | Na.K | -0.602 | 0.014 |
| TH | CAT | 0.191 | 0.479 | MDA | Na.K | 0.621 | 0.010 |
| TCSA | CAT | 0.293 | 0.271 | H_2_O_2_ | Na.K | 0.384 | 0.142 |
| CV | CAT | -0.201 | 0.456 | Prol | Na.K | 0.726 | 0.001 |
| TC | CAT | -0.070 | 0.796 | APX | Na.K | 0.942 | 0.000 |
| MDA | CAT | 0.944 | 0.000 | POX | Na.K | 0.627 | 0.009 |
| H_2_O_2_ | CAT | 0.962 | 0.000 | CAT | Na.K | 0.575 | 0.020 |
| Prol | CAT | 0.639 | 0.008 | SOD | Na.K | 0.637 | 0.008 |
| APX | CAT | 0.662 | 0.005 | Na | Na.K | 0.851 | 0.000 |
| POX | CAT | 0.017 | 0.949 | K | Na.K | -0.818 | 0.000 |
| TH | SOD | 0.022 | 0.936 |  |  |  |  |

Abbreviations: As in Supplementary Table 2.

**Table S4** Pearson's correlation coefficients (*r*) and the associated p-values (*p*) between the measured traits in peach.

| Trait | Trait | *r* | *p* | Trait | Trait | *r* | *p* |
| --- | --- | --- | --- | --- | --- | --- | --- |
| TH | TCSA | 0.718 | 0.002 | TCSA | SOD | -0.055 | 0.840 |
| TH | CV | 0.933 | 0.000 | CV | SOD | -0.504 | 0.046 |
| TCSA | CV | 0.827 | 0.000 | TC | SOD | -0.815 | 0.000 |
| TH | TC | 0.585 | 0.017 | MDA | SOD | 0.559 | 0.024 |
| TCSA | TC | 0.043 | 0.873 | H_2_O_2_ | SOD | 0.881 | 0.000 |
| CV | TC | 0.363 | 0.167 | Prol | SOD | 0.781 | 0.000 |
| TH | MDA | -0.915 | 0.000 | APX | SOD | -0.328 | 0.215 |
| TCSA | MDA | -0.695 | 0.003 | POX | SOD | -0.401 | 0.123 |
| CV | MDA | -0.926 | 0.000 | CAT | SOD | 0.830 | 0.000 |
| TC | MDA | -0.449 | 0.081 | TH | Na | -0.880 | 0.000 |
| TH | H_2_O_2_ | -0.819 | 0.000 | TCSA | Na | -0.910 | 0.000 |
| TCSA | H_2_O_2_ | -0.351 | 0.182 | CV | Na | -0.970 | 0.000 |
| CV | H_2_O_2_ | -0.729 | 0.001 | TC | Na | -0.278 | 0.298 |
| TC | H_2_O_2_ | -0.815 | 0.000 | MDA | Na | 0.892 | 0.000 |
| MDA | H_2_O_2_ | 0.760 | 0.001 | H_2_O_2_ | Na | 0.640 | 0.008 |
| TH | Prol | -0.927 | 0.000 | Prol | Na | 0.821 | 0.000 |
| TCSA | Prol | -0.566 | 0.022 | APX | Na | 0.696 | 0.003 |
| CV | Prol | -0.886 | 0.000 | POX | Na | 0.641 | 0.008 |
| TC | Prol | -0.665 | 0.005 | CAT | Na | -0.105 | 0.700 |
| MDA | Prol | 0.870 | 0.000 | SOD | Na | 0.362 | 0.168 |
| H_2_O_2_ | Prol | 0.930 | 0.000 | TH | K | 0.301 | 0.258 |
| TH | APX | -0.387 | 0.138 | TCSA | K | -0.276 | 0.301 |
| TCSA | APX | -0.801 | 0.000 | CV | K | 0.088 | 0.746 |
| CV | APX | -0.603 | 0.013 | TC | K | 0.887 | 0.000 |
| TC | APX | 0.471 | 0.065 | MDA | K | -0.165 | 0.542 |
| MDA | APX | 0.490 | 0.054 | H_2_O_2_ | K | -0.655 | 0.006 |
| H_2_O_2_ | APX | -0.043 | 0.874 | Prol | K | -0.431 | 0.095 |
| Prol | APX | 0.240 | 0.372 | APX | K | 0.732 | 0.001 |
| TH | POX | -0.315 | 0.235 | POX | K | 0.791 | 0.000 |
| TCSA | POX | -0.749 | 0.001 | CAT | K | -0.987 | 0.000 |
| CV | POX | -0.531 | 0.034 | SOD | K | -0.839 | 0.000 |
| TC | POX | 0.521 | 0.038 | Na | K | 0.046 | 0.865 |
| MDA | POX | 0.427 | 0.099 | TH | Na.K | -0.678 | 0.004 |
| H_2_O_2_ | POX | -0.107 | 0.693 | TCSA | Na.K | -0.030 | 0.912 |
| Prol | POX | 0.183 | 0.498 | CV | Na.K | -0.527 | 0.036 |
| APX | POX | 0.990 | 0.000 | TC | Na.K | -0.843 | 0.000 |
| TH | CAT | -0.244 | 0.362 | MDA | Na.K | 0.615 | 0.011 |
| TCSA | CAT | 0.347 | 0.188 | H_2_O_2_ | Na.K | 0.896 | 0.000 |
| CV | CAT | -0.027 | 0.920 | Prol | Na.K | 0.814 | 0.000 |
| TC | CAT | -0.871 | 0.000 | APX | Na.K | -0.302 | 0.255 |
| MDA | CAT | 0.124 | 0.647 | POX | Na.K | -0.356 | 0.176 |
| H_2_O_2_ | CAT | 0.640 | 0.008 | CAT | Na.K | 0.795 | 0.000 |
| Prol | CAT | 0.408 | 0.117 | SOD | Na.K | 0.949 | 0.000 |
| APX | CAT | -0.775 | 0.000 | Na | Na.K | 0.384 | 0.142 |
| POX | CAT | -0.819 | 0.000 | K | Na.K | -0.800 | 0.000 |
| TH | SOD | -0.622 | 0.010 |  |  |  |  |

Abbreviations: As in Supplementary Table 2.
