# Supplementary material for: Sodicity stress differently influences physiological traits and anti-oxidant enzymes in pear and peach cultivars
Source: PeerJ. 2023 Mar 13;11:e14947. doi: 10.7717/peerj.14947 (PMC10019333; doi:10.7717/peerj.14947)
Supplement: Supplemental Information 2 [file peerj-11-14947-s002.docx]

Raw_data for different traits in pear

| **Trt** | **Cultivar** | **TH** | **TCSA** | **CV** | **TC** | **MDA** | **H2O2** | **Prol** | **APX** | **POX** | **CAT** | **SOD** | **Na** | **K** | **Na/K** |
| --- | --- | --- | --- | --- | --- | --- | --- | --- | --- | --- | --- | --- | --- | --- | --- |
| Control | Punjab Beauty | 3.65 | 35.21 | 2.02 | 1.28 | 8.65 | 130.44 | 4.37 | 14.77 | 185.43 | 6.05 | 66.3 | 0.82 | 9.01 | 0.09 |
| Control | Punjab Beauty | 4.13 | 33.88 | 1.61 | 1.53 | 9.25 | 128.18 | 3.42 | 13.65 | 189.7 | 6.64 | 64.13 | 0.74 | 10.21 | 0.07 |
| Control | Punjab Beauty | 3.81 | 38.55 | 2.08 | 1.41 | 8.92 | 133.01 | 3.5 | 14.29 | 175.9 | 5.92 | 61.85 | 0.91 | 10 | 0.09 |
| Control | Punjab Beauty | 3.85 | 39.38 | 1.92 | 1.43 | 8.98 | 130.02 | 3.82 | 13.69 | 184 | 6.17 | 63.98 | 0.84 | 9.81 | 0.09 |
| Sodic | Punjab Beauty | 3.35 | 27.89 | 1.11 | 0.93 | 10.27 | 142.97 | 5.09 | 19.13 | 217.29 | 7.45 | 86.07 | 1.1 | 8.09 | 0.14 |
| Sodic | Punjab Beauty | 3.24 | 33.25 | 1.28 | 1.16 | 9.74 | 150.64 | 5.35 | 17.36 | 226.43 | 7.99 | 75.34 | 1.2 | 8.29 | 0.14 |
| Sodic | Punjab Beauty | 3.07 | 29.02 | 0.81 | 1.03 | 9.42 | 145.12 | 4.74 | 17.85 | 228.5 | 7.85 | 80.45 | 1.24 | 7.63 | 0.16 |
| Sodic | Punjab Beauty | 3.25 | 32.62 | 1.05 | 1.01 | 9.79 | 146.33 | 5.04 | 18.09 | 223.88 | 7.71 | 81.04 | 1.16 | 7.95 | 0.15 |
| Control | Patharnakh | 3.05 | 25.98 | 2.3 | 1.07 | 7.15 | 102.93 | 4.19 | 14.37 | 224.85 | 4.5 | 56.59 | 0.53 | 8.36 | 0.06 |
| Control | Patharnakh | 2.84 | 23.75 | 1.15 | 1.15 | 7.67 | 103.76 | 3.54 | 12.74 | 220.15 | 4.15 | 54.7 | 0.64 | 9 | 0.07 |
| Control | Patharnakh | 2.91 | 21.39 | 1.19 | 1.18 | 7.5 | 112.18 | 4.06 | 13.38 | 216.4 | 4.61 | 57.4 | 0.59 | 8.27 | 0.07 |
| Control | Patharnakh | 3.3 | 23.61 | 1.47 | 1.09 | 7.39 | 104.08 | 3.88 | 13.66 | 220.53 | 4.48 | 56.43 | 0.62 | 8.61 | 0.06 |
| Sodic | Patharnakh | 2.24 | 15.97 | 0.5 | 0.79 | 9.81 | 118.82 | 4.57 | 18.62 | 240.66 | 5.71 | 62.48 | 0.95 | 6.1 | 0.16 |
| Sodic | Patharnakh | 1.98 | 13.13 | 0.55 | 0.76 | 8.13 | 121.42 | 5.13 | 16.96 | 233.76 | 6.24 | 69.7 | 1.07 | 6.63 | 0.16 |
| Sodic | Patharnakh | 2.24 | 12.14 | 0.37 | 1.07 | 8.76 | 115.48 | 4.42 | 18.9 | 242.1 | 6.07 | 68.35 | 1.09 | 5.54 | 0.20 |
| Sodic | Patharnakh | 2.09 | 13.8 | 0.45 | 0.91 | 8.93 | 118.47 | 4.7 | 18.34 | 239.05 | 5.95 | 67.02 | 0.99 | 6.11 | 0.18 |

Raw_data for different traits in peach

| **Trt** | **Cultivar** | **TH** | **TCSA** | **CV** | **TC** | **MDA** | **H2O2** | **Prol** | **APX** | **POX** | **CAT** | **SOD** | **Na** | **K** | **Na/K** |
| --- | --- | --- | --- | --- | --- | --- | --- | --- | --- | --- | --- | --- | --- | --- | --- |
| Control | Partap | 4.48 | 236.3 | 24.87 | 1.65 | 7.34 | 143.86 | 4.08 | 23.83 | 308.62 | 3.89 | 48.25 | 1.21 | 13.67 | 0.09 |
| Control | Partap | 4.55 | 239.58 | 30.34 | 1.58 | 8.19 | 133.52 | 3.89 | 20.45 | 301.4 | 4.16 | 47.3 | 1.1 | 14.52 | 0.08 |
| Control | Partap | 3.92 | 217.75 | 31.52 | 1.41 | 7.59 | 137.15 | 4.14 | 22.14 | 312.2 | 3.75 | 45.1 | 1.08 | 15.07 | 0.07 |
| Control | Partap | 4.06 | 231.04 | 29.12 | 1.61 | 7.82 | 137.96 | 4.11 | 22.18 | 308.02 | 3.91 | 46.94 | 1.11 | 14.37 | 0.08 |
| Sodic | Partap | 2.66 | 56.58 | 2.78 | 1.27 | 9.56 | 163.23 | 5.05 | 26.55 | 320.64 | 5.14 | 56.13 | 1.84 | 9.83 | 0.19 |
| Sodic | Partap | 2.59 | 58.64 | 3.03 | 0.91 | 11.82 | 182.76 | 5.32 | 23.16 | 325.75 | 5.29 | 51.36 | 2.01 | 10.09 | 0.20 |
| Sodic | Partap | 2.84 | 62.87 | 2.59 | 1.08 | 10.29 | 180.75 | 5.49 | 25.84 | 333.4 | 5.24 | 55.9 | 1.98 | 9.51 | 0.21 |
| Sodic | Partap | 2.75 | 59.71 | 2.78 | 1.04 | 10.63 | 175.54 | 5.36 | 24.99 | 327.04 | 5.31 | 54.66 | 1.88 | 9.77 | 0.19 |
| Control | Shan-e-Punjab | 4.48 | 273.53 | 38.57 | 1.06 | 6.92 | 150.89 | 4.12 | 11.61 | 185.67 | 6.94 | 53.18 | 0.96 | 6.01 | 0.16 |
| Control | Shan-e-Punjab | 4.28 | 255.13 | 32.34 | 1.11 | 6.98 | 166.29 | 4.38 | 12.64 | 177.4 | 7.03 | 54.07 | 0.91 | 5.88 | 0.15 |
| Control | Shan-e-Punjab | 4.13 | 283.98 | 31.75 | 0.98 | 7.18 | 144.36 | 3.93 | 10.88 | 171.6 | 6.81 | 51.74 | 0.88 | 5.21 | 0.17 |
| Control | Shan-e-Punjab | 4.34 | 268.23 | 34.44 | 1.03 | 7.08 | 154.02 | 4.22 | 13.13 | 179.04 | 6.88 | 53.41 | 0.93 | 5.74 | 0.16 |
| Sodic | Shan-e-Punjab | 3.08 | 253.21 | 16.23 | 0.92 | 9.94 | 181.95 | 4.79 | 16.78 | 211.92 | 7.29 | 60.12 | 1.35 | 4.24 | 0.32 |
| Sodic | Shan-e-Punjab | 2.8 | 230.08 | 12.89 | 0.87 | 9.21 | 187.6 | 5.75 | 14.32 | 220.04 | 7.54 | 59.95 | 1.45 | 4.07 | 0.36 |
| Sodic | Shan-e-Punjab | 3.22 | 231.69 | 12.76 | 0.81 | 9.76 | 192.53 | 5.84 | 15.67 | 225.7 | 7.71 | 62.45 | 1.52 | 4.65 | 0.33 |
| Sodic | Shan-e-Punjab | 3.09 | 236.87 | 14.93 | 0.85 | 11.07 | 187.25 | 5.16 | 15.71 | 220.17 | 7.63 | 61.02 | 1.4 | 4.28 | 0.33 |
